# Chromogranin A and Its Fragments as Regulators of Small Intestinal Neuroendocrine Neoplasm Proliferation 

**DOI:** 10.1371/journal.pone.0081111

**Published:** 2013-11-19

**Authors:** Francesco Giovinazzo, Simon Schimmack, Bernhard Svejda, Daniele Alaimo, Roswitha Pfragner, Irvin Modlin, Mark Kidd

**Affiliations:** 1 Department of Surgery, Yale University School of Medicine, New Haven, Connecticut, United States of America; 2 Laboratory of Translational Surgery-LURM, University of Verona, Verona, Italy; 3 University Hospital of General, Visceral- and Transplantation-Surgery of Heidelberg, Heidelberg, Germany; 4 Department of Pathophysiology and Immunology, University of Graz, Graz, Austria; Institute of Hepatology - Birkbeck, University of London, United Kingdom

## Abstract

**Introduction:**

Chromogranin A is a neuroendocrine secretory product and its loss is a feature of malignant NEN de-differentiation. We hypothesized that chromogranin A fragments were differentially expressed during NEN metastasis and played a role in the regulation of NEN proliferation.

**Methods:**

Chromogranin A mRNA (PCR) and protein (ELISA/western blot) were studied in 10 normal human mucosa, 5 enterochromaffin cell preparations, 26 small intestinal NEN primaries and 9 liver metastases. Cell viability (WST-1 assay), proliferation (bromodeoxyuridine ELISA) and expression of AKT/AKT-P (CASE ELISA/western blot) in response to chromogranin A silencing, inhibition of prohormone convertase and mTOR inhibition (RAD001/AKT antisense) as well as different chromogranin A fragments were examined in 4 SI-NEN cell lines.

**Results:**

Chromogranin A mRNA and protein levels were increased (37-340 fold, *p*<0.0001) in small intestinal NENs compared to normal enterochromaffin cells. Western blot identified chromogranin A-associated processing bands including vasostatin in small intestinal NENs as well as up-regulated expression of prohormone convertase in metastases. Proliferation in small intestinal NEN cell lines was decreased by silencing chromogranin A as well as by inhibition of prohormone convertase (*p*<0.05). This inhibition also decreased secretion of chromogranin A (*p*<0.05) and 5-HT (*p*<0.05) as well as expression of vasostatin. Metastatic small intestinal NEN cell lines were stimulated (50-80%, *p*<0.05) and AKT phosphorylated (Ser473: *p*<0.05) by vasostatin I, which was completely reversed by RAD001 (*p*<0.01) and AKT antisense (*p*<0.05) while chromostatin inhibited proliferation (~50%, *p*<0.05).

**Conclusion:**

Chromogranin A was differentially regulated in primary and metastatic small intestinal NENs and cell lines. Chromogranin A fragments regulated metastatic small intestinal NEN proliferation via the AKT pathway indicating that CgA plays a far more complex role in the biology of these tumors than previously considered.

## Introduction

Chromogranin A (CgA), a heat stable, hydrophilic acidic protein of ~460 amino acids, is a member of the granin family of secretory proteins that are ubiquitous to the nervous, endocrine and immune system [1,2]. It forms the principal component of the soluble core of dense-core secretory granules in neuroendocrine cells and is secreted from these cells in a physiologically regulated manner [3]. The biosynthesis of CgA can be influenced both at a transcriptional level and post-translationally [4]. CgA is elevated in a number of pathological conditions e.g. renal failure [5] and, irrespective of the mechanism of processing, its peptides have been proposed to regulate a range of physiological processes [6], an example being colonic motility [7]. Transcripts [8,9] and plasma levels of the protein are specifically elevated in patients with different endocrine tumors such as pheochromocytomas and medullary thyroid carcinoma, as well as in bronchopulmonary and gastroenteropancreatic neuroendocrine neoplasms (GEP-NENs) [1,2].

**Figure 1:**
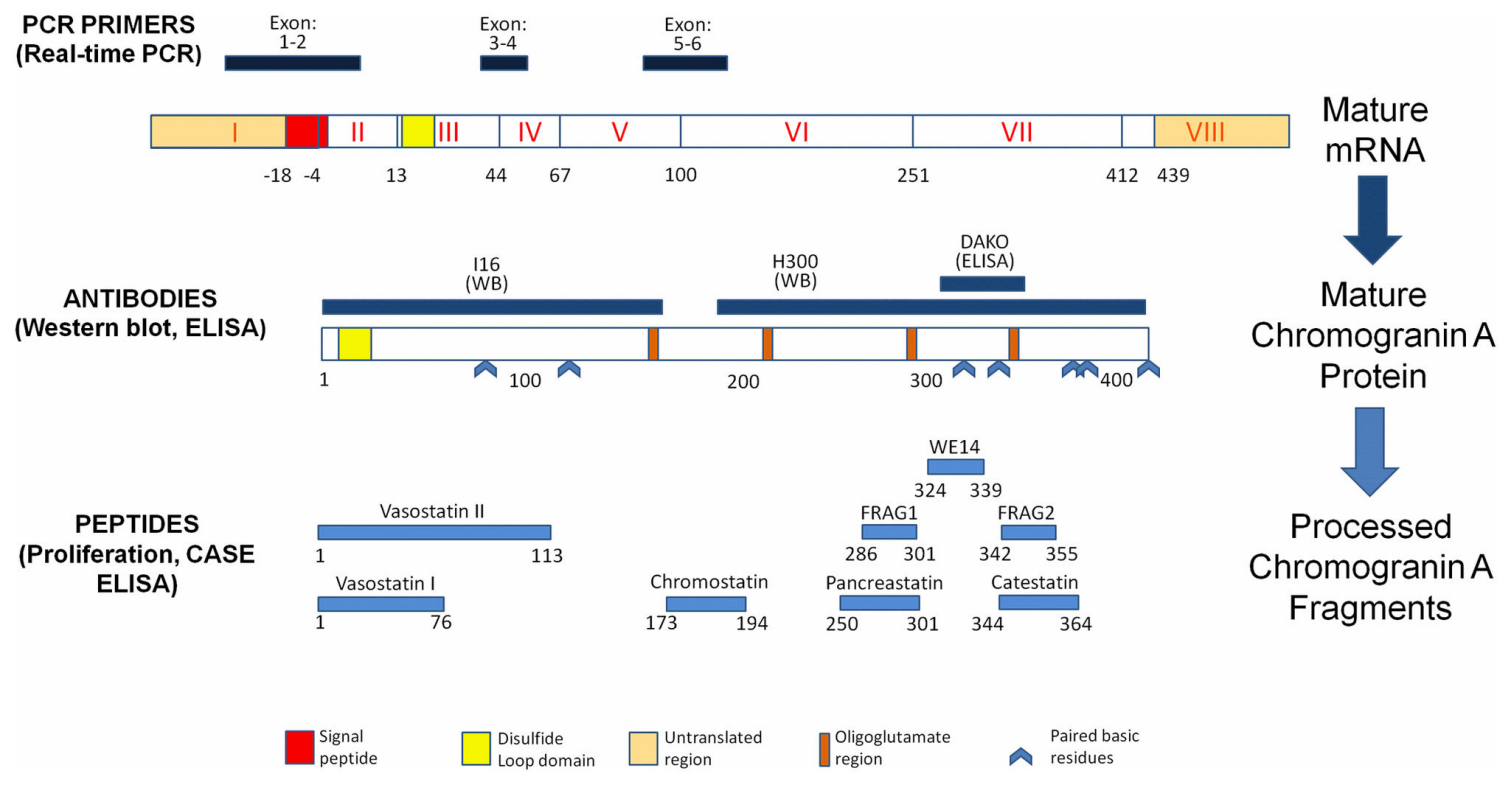
CgA coding regions, putative functional domains and targets. The primary transcript, on chromosome 14, is derived from 8 exons and includes exon 1 which is untranslated but contains a signal peptide region for protein processing. In this study, PCR was performed using intron spanning primers to examine exons I-VI. Mature CgA mRNA includes 439 coding base pairs which are translated into a primary peptide of 431 amino acids. Processing of CgA following cleavage at dibasic and monobasic residues e.g. by PC1/3 and CPE results in production of a range of intermediate peptides as well biologically active peptides [36].

Western blot identifies a range of CgA fragment sizes from ~9-85 kDa depending on processing, neuroendocrine cell type and antibody used [10], but the parent CgA molecule is generally considered to have a molecular mass of ~70-85 kDa [11]. Smaller fragments reflects post-translational processing and the production of a series of smaller biologically active peptides such as vasostatin I and II (corresponding to CgA residues 1-76 and 1-113, respectively), chromostatin (CgA 173-194), pancreastatin (CgA 250-301), WE14 (CgA 324-337) and catestatin (CgA 344-372) [1,2,12] (Figure **1**). Post-transcription (translational) modifications are regulated by a number of enzymes. These include the serine protease prohormone convertase 1-3 (PC1-3) [13], which is associated with production of pancreastatin [14], the cysteine protease cathepsin L [15], associated with production of the middle and C-terminal fragments e.g. catestatin, or by the fibrinolytic enzyme, plasmin [16].

Although CgA is a well-characterized product of NENs, very few studies have investigated the role of either CgA or its cleavage fragments as regulators of small intestinal NEN (SI-NEN) proliferation, the commonest tumor in this class [17,18]. Given the differences in transcription and processing of CgA in different neuroendocrine tissues and their neoplasia [1,2], e.g. SI-NENs with liver metastases [19], we hypothesized that CgA transcripts were differentially expressed during NEN metastasis, that this translated into differences in CgA fragment expression and that these specific fragments may regulate NEN proliferation. 

We specifically focused on SI-NENs since these are common and there are a number of well-characterized cell lines available [20-24]. Initially, we examined mRNA and protein expression in normal mucosa and tumor tissue samples, and then measured proliferation in four tumor cell lines, two primary tumor-derived lines, KRJ-I and P-STS [20,24], and two metastases, L-STS and H-STS [24]. As proliferation of tumors is related to AKT/mTOR activation and signaling and can be inhibited by rapamycin-derivatives [20], we specifically evaluated the effects of candidate peptides on this pathway as well as on tumor cell proliferation. To characterize the role of post-translational effectors, we evaluated mRNA and protein expression of the CgA processing enzyme prohormone convertase in both tumor tissue samples as well as cell lines and evaluated the effect of proliferation on CgA and this processing enzyme *in vitro*. We also examined the effect of CgA silencing and pharmacologic inhibition of prohormone convertase on tumor cell proliferation, secretion and post-transcriptional changes in CgA fragment expression. The results identified a role for CgA peptides in the regulation of NEN proliferation at the level of AKT signaling. Targeting AKT or prohormone convertases specifically decreased proliferation, especially in metastases.

## Materials and Methods

### Small intestinal neuroendocrine neoplasm tissue and cell lines

In total, 10 samples of normal small intestinal mucosa, 5 preparations of normal human enterochromaffin (EC) cells, obtained from fluorescence-activated cell sorting of normal mucosa; >98% pure EC cells [25]), 14 primary SI-NENs (localized – no evidence of metastases), 12 primary SI-NENs (with evidence of distant metastases) and 9 corresponding liver metastases were collected for real-time PCR analysis, western blot and ELISA analysis. All tumors contained >80% pure neoplastic cells and were tryptophan hydroxylase1-positive and therefore EC cell-derived [25]). All SI-NENs were classified pathologically according to the WHO standard 2006 as well differentiated neuroendocrine tumors (WDNETs, *n*=26), now classified as G1 NETs [26]. All samples were collected and analyzed according to an IRB protocol (Yale University School of Medicine). The protocol was specifically approved for this study. Written consent was obtained from all study participants.

To evaluate the biological function of the candidate CgA fragments (Figure **1** and Table **1**), four well-characterized NEN cell lines (KRJ-I and P-STS: primary tumors, L-STS: lymph node metastasis, and H-STS: hepatic metastasis) were cultured in Quantum 263 complete tumor medium (PAA, Dartmouth, MA) supplemented with 100 IU penicillin/ml and 100 µg streptomycin/ml at 37°C with 5% CO_2_. During growth phases, 40-90% of e.g., KRJ-1 cells were Ki67 positive [20-24] reflecting *in vitro* growth characteristics. While not commensurate with *in vivo* SI-NEN behavior (Ki67<20%), these provide robust, well-characterized models for assessing proliferation. All experiments were performed without antibiotics. 

**Table 1:** Peptide fragments used for the *in vitro* proliferation studies.

**CgA fragment peptides**					
	**Position**	**Position**	**Size** (**kDa**)	**Source**	
**Vasostatin I**	N-terminal	1-76	8.5 kDa	Phoenix	
**Vasostatin II**	N-terminal	1-133	12.8 kDa	Phoenix	
**Pancreastatin**	Middle	250-301	4.2 kDa	Phoenix	
**Chromostatin**	C-terminal	173-194	2.0 kDa	Phoenix	
**Fragment 1**	C-terminal	286-301	1.7 kDa	Phoenix	
**Fragment 2**	C-terminal	342-355	1.4 kDa	Phoenix	
**WE14**	C-terminal	324-337	1.6 kDa	N E Peptides	
**Catestatin**	C-terminal	352-372	2.2 kDa	N E Peptides	

N E Peptides = New England Peptides

### RNA isolation and real-time polymerase chain reaction

Messenger RNA was extracted and converted to cDNA from small pieces (~20mg) of tissue or cell line lysates (1x10^6^ cells) as described [27] using TRIZOL^®^ (Invitrogen, Carlsbad, CA) and the High Capacity cDNA Archive Kit (Applied Biosystems, Carlsbad, CA). Transcript levels of *CgA* (exons 1-6; corresponding amino acids 1-251 and >85% of the coding region; see Figure **1**) and prohormone convertase (*PCSK1*) expression were quantified using Assays-on-Demand^TM^ products and the ABI 7900 Sequence Detection System (both Applied Biosystems) according to the manufacturers’ instructions. PCR data were normalized to the housekeeping gene, ALG9 (asparagine linked glycosylation 9) [28] using the C_T_ method [29].

### Protein isolation, ELISA and Western blot analysis

Small pieces (~20mg) of tissue or cell line lysates (from 1x10^6^ cells) were processed as described [27] including manual homogenization with RIPA lysis buffer (Millipore, Temecula, CA) with addition of complete protease inhibitor (Roche, Indianapolis, IN), phosphatase inhibitor sets 1 and 2 (Calbiochem, La Jolla, CA), 100 mM phenylmethylsulfonylfluoride (Roche), 200 mM Na3VO4 (Acros Organics,Geel, Belgium), and 12.5 mg/ml sodium dodecyl sulfate (American Bioanalytical, Natick, MA) and protein quantification undertaken using the Pierce BCA protein assay (Thermo Scientific, Rockford, IL). 

CgA protein levels were firstly quantified via ELISA using a commercially available kit (DAKO, K0025, Glostrup, Denmark) as described in the manufacturer’s instructions. Final results were normalized to protein levels and expressed as Units CgA/µg protein for samples and cell lines. For western blot analysis, total protein lysates (15µg) were denatured in SDS sample buffer (Invitrogen), separated on an SDS-PAGE gel (10%, Invitrogen) and transferred to a PVDF membrane with a pore size of 0.45 mm (Bio-Rad, Hercules, CA). After blocking (5% bovine serum albumin [BSA]) for 60 min at room temperature (RT), the membrane was incubated with either anti-CgA (complete protein, DAK-A3, 1:200) or anti-prohormone convertase 1-3 (Abcam, Boston, MA, 1:1000) and separately with anti-β-actin (Sigma-Aldrich, St. Louis, MO, 1:10,000) antibodies in 5% BSA/PBS/Tween 20 overnight at 4°C. After washing in PBS/Tween 20, the membranes were incubated with the horseradish peroxidase-conjugated secondary antibodies (Cell Signaling, Danvers, MA) for 60 min at RT. After washing, immunodetection was performed using the Supersignal West Pico Luminol/ Enhancer solution (Thermo Scientific). Protein expression in cell lines was reported relative to β-actin (Sigma-Aldrich).

### Effect of proliferation on CgA and processing enzymes

H-STS cells were sub-cultured and collected at days 2, 3 and 7, which are time points during logarithmic (~70%) and plateau (~30%) growth curves [20,21,24]. PCR and western blot were undertaken on CgA (exons 1-6) and prohormone convertases 1-3, respectively. Transcript and protein results were compared to day 2 (logarithmic growth) [20,21,24].

### Effect of CgA, its peptides and processing enzymes on proliferation and secretion

In order to investigate the role of CgA in tumor cell proliferation, 2 x 10^5^ H-STS cells/well were seeded in 12-well plates (Falcon, BD, Franklin Lakes, NJ) and CgA silenced using the reverse transfection approach with siRNA (Invitrogen, 40 pmol/well, sense sequence: GCUACAAGGAGAUCCGGAA) and Lipofectamine 2000 (Invitrogen) in comparison to scrambled siRNA (Invitrogen) as a control. We confirmed the knockdown using PCR after 48 and 96 hours of incubation and performed BrdU (Bromodeoxyuridine) ELISA (Roche Diagnostics, Indianapolis, IN) according to the manufacturers’ instructions. Briefly, after silencing H-STS cells in 96-well plates (clear bottom, Costar, Corning, NY), they were labeled with BrdU uptake solution and incubated for 3 hrs. Cells were fixed, DNA denatured and anti-BrdU antibody solution added. After 90 min incubation, the final substrate was added and the chemiluminescence read on a GLOMAX Luminometer (Promega, Madison, WI). Luminescence (relative values) in CgA knocked down cells were compared to scrambled siRNA/Lipofectamine treated cells and represented as a fold-decrease.

A complementary approach to investigate the role of translational processing on CgA was undertaken by inhibiting PC activity. H-STS cells were seeded in either 6-well (1 x 10^6^ cells/well) or 96-well plates (2 x 10^4^ cells/well) and treated with the prohormone convertase inhibitor [30] decanoyl-Arg-Val-Lys-Arg-chloromethylketone (Millipore, Billercia, MA, 25 and 50µM) for 48 hrs. Western blot was used to evaluate alterations in CgA processing while effects on proliferation were assessed by BrdU-ELISA. 

To confirm a functional effect of inhibiting CgA itself or its processing by either silencing CgA or inhibition of CgA processing enzyme prohormone convertase on cells [31], CgA and serotonin secretion was evaluated at the termination of the experiment (48 hrs) in the supernatant (ELISA: Labor Diagnostika, Nordhorn, Germany) [32].

To examine the effect of processed CgA peptides on proliferation, 2 x 10^5^ cells/ml were seeded in 96 well plates (Falcon, BD, Franklin Lakes, NJ) at 100 µl and stimulated after 2 days with different fragments (Phoenix Pharmaceuticals and New England Peptides, Gardner, MA) (see Table **1**, concentration 10^-9^M to 10^-6^M) for 72 hrs in comparison to no treatment (control) and to pre-incubation with the mTOR inhibitor everolimus (RAD001, 10^-9^M) for 30 min prior to application of the peptide [33]. Cell viability was analyzed using the WST1 cell proliferation reagent (Roche) according to the manufacturers’ instructions [34]. Optical density was quantified spectrophotometrically at 450nm. Results were normalized to the unstimulated control and effective half-maximal concentration (EC_50_ or IC_50_) was calculated [21,33].

### AKT/ mTOR pathway activation

H-STS was stimulated with CgA fragments vasostatin I and P-STS with chromostatin (10^-6^ M) for 1 hr. One cell line set were pre-incubated for 30 min with RAD001 (10^-9^M) [33] or AKT antisense oligonucleotide (TCCCTCTTGCTCGTGTTCC, Yale Medical School Keck Oligonucleotide Synthesis Facility) [35] prior to application of the peptide. AKT signal activity (pAKT) was determined using SuperArray CASE^TM^ ELISA kits (AKT FE-001, Biomol, Hamburg, Germany) and western blot using anti-pAKT (Ser473, Cell Signaling) as described [20,33]. BrdU uptake was measured to evaluate the effect of AKT inhibition on vasostatin or chromostatin-regulated proliferation in each of the cell lines.

### Identification of a candidate “receptor” for CgA

To identify potential CgA receptors, we examined expression of Cy5-labeled CgA (342-355, Phoenix Pharmaceuticals, Burlingame, CA) fluorescence in KRJ-I cells and H-STS cells using confocal microscopy as described [20].

### Statistical Evaluation

All statistical analyses were performed using Microsoft Excel and Prism 5 (GraphPad Software, San Diego, CA). Sigmoidal dose responses and nonlinear regression analyses were calculated to identify the EC_50_ and IC_50_ concentrations for each agent. Alterations in signal transduction and transcriptional activation were assessed using 2-tailed Wilcoxan rank sum tests for non-parametric data. Multiple group comparisons were performed using the Kruskal Wallis test, followed by the Dunn’s post-hoc test where appropriate. A *p*-value of <0.05 was designated as significant. 

## Results

### CgA mRNA and protein expression in small intestinal NENs and cell lines

**Figure 2:**
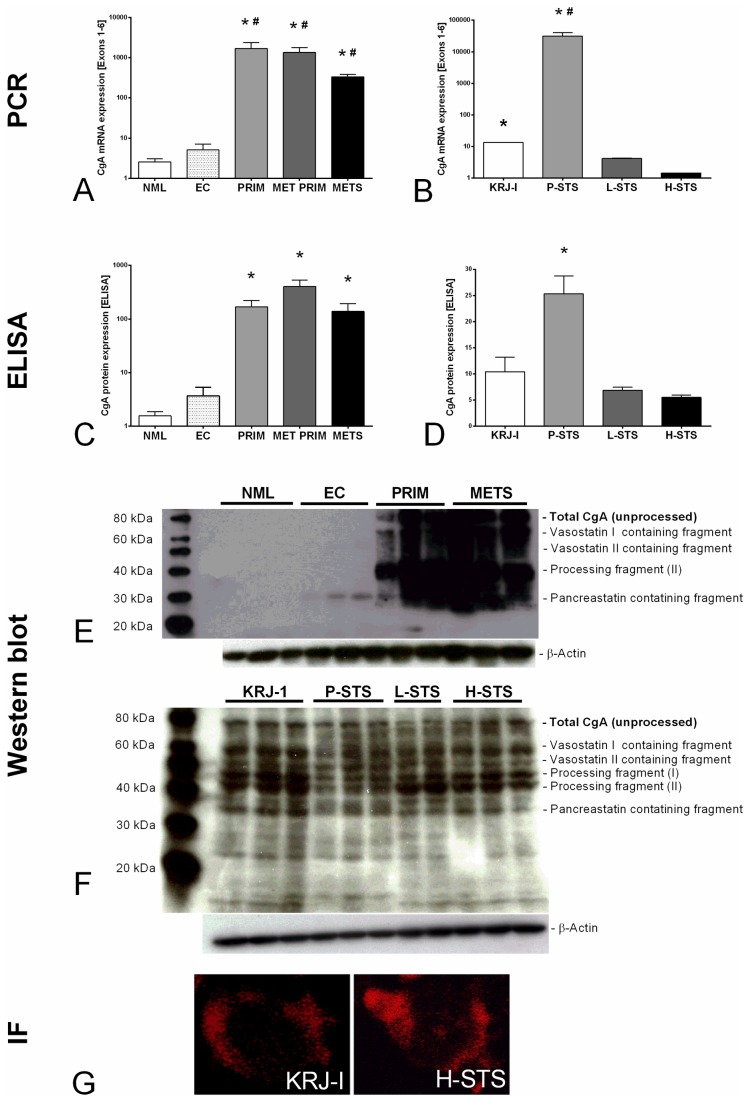
Chromogranin A expression in normal mucosa, EC cells, small intestinal NENs (SI-NENs) and primary and metastatic SI-NEN cell lines. CgA mRNA expression in normal human mucosa (NML), EC cell preparations (EC), localized NENs (PRIM), primaries with metastasis (MET PRIM) and liver metastases (METS) demonstrated that all NENs expressed higher CgA levels (Kruskal-Wallis *p*<0.0001) compared to normal mucosa (*p<0.001) or normal EC cells (^#^ *p*<0.001) (**a**). Levels of CgA protein expression, measured by ELISA, showed a similar pattern (**2C**, Kruskal-Wallis *p*<0.0001) and was increased in PRIM (*p<0.01), MET PRIM (*p<0.001) and METS (*p<0.05) compared to normal mucosa. CgA western blot in normal mucosa, normal EC cells and SI-NENs identified a mature CgA band of 75-80 kDa in all NENs but not in normal mucosa or EC cells (**2E**). Fragment sizes included peptides ranging in size from ~30-60kDa, consistent with CgA processing intermediates [36]. In cell lines, CgA mRNA was expressed in higher levels in the two primary cell lines in comparison to metastatic cell lines (**2B**, Kruskal-Wallis *p*<0.0001), particularly in P-STS CgA was over-expressed compared to H-STS (* *p*<0.001) and L-STS cells (^#^ *p*<0.01). In KRJ-1 CgA was also elevated in comparison to H-STS cells (*p<0.01, **2B**). Protein level (ELISA) followed similar pattern (Kruskal-Wallis *p*=0.0273, *p<0.05, **2D**). Using western blot, total CgA (75-80 kDa) was identified in all cell lines, highest in the primary cell lines KRJ-1 and P-STS (2F). Band sizes consistent with CgA processing were evident and exhibited different patterns of expression consistent with alterations in translational modifications (2F). No external receptor was identified for CgA, but Cy5-labeled immunofluorescence (IF) was identified within KRJ-I and H-STS cells. We interpret this dot-like signal to reflect intracellular uptake of this CgA peptide (**2G**).

Messenger RNA levels of all CgA exons that were examined (exons 1-6) were increased in primary NENs with or without metastases as well as in metastases compared to normal mucosa (540-680x, (*p*<0.0001) and normal EC cell preparations (270-340x, *p*<0.001) (Figure **2A**). Transcripts tended to be lower in metastases than in the primaries suggesting an alteration in transcriptional regulation in these tumors. 

We next examined CgA protein expression in these samples using ELISA (Figure **2C**). The reproducibility of this assay (inter-assay and intra-assay coefficients of variability) for mucosal tissue was 7 and 5%, respectively. Normal mucosa expressed 1.6±0.3 Units CgA/µg protein, normal EC cells 3.7±1.6 Units CgA/µg protein. Levels in neoplasms ranged from 168.6±53 (localized primaries) to 402.2±132.4 Units/µg protein in neoplasms with metastases (0.42-fold). CgA protein levels were significantly increased in SI-NENs compared to normal mucosa [vs. localized primaries *p*<0.01 (105-fold), vs. metastasized primaries *p*<0.001 (251-fold) and vs. metastases *p*<0.05 (86-fold)] as well as compared to EC cell preparations (*p*<0.01) (Figure **2C**). Protein levels in metastases (138.4±54.9 Units/µg protein) tended to be lower than in the localized tumors that exhibited metastases (0.33-fold), consistent with the mRNA results (Figure **2A**, 334.8 vs. 1353, 0.25 fold).

Using a western blot approach to examine expression of different CgA fragments, predominant bands of ~76-80 kDa were noted for CgA in all NEN samples but not in normal mucosa or in normal EC cell preparations (Figure **2E**), confirming over-expression of CgA and its fragments in neuroendocrine neoplastic tissue. Processing of CgA was evident in all SI-NEN samples. In particular, band sizes consistent with intermediate fragments including Vasostatin I and II [36] (confirmed by a Vasostatin I/II antibody [sc-23556, Santa Cruz, CA], *data not shown*) were highly expressed in metastases. Other processing fragments as well as fragments that included pancreastatin were also identified in neoplasia; levels were increased in comparison to normal EC cells (Figure **2E**).

Expression of CgA mRNA in the two primary SI-NEN cell lines (KRJ-1 and P-STS) was also elevated compared to normal EC cells (3-6000 fold), while the two metastatic cell lines (L-STS and H-STS) expressed lower levels of CgA mRNA in comparison to normal EC cells (0.08-0.3 fold). Both primary cell lines expressed higher levels of CgA mRNA than the metastatic cell lines (Figure **2B**, Kruskal-Wallis *p*<0.0001 in all exons). CgA was elevated, particularly in P-STS, compared to H-STS (*p*<0.001) and L-STS cells (*p*<0.01); CgA, likewise, was higher in KRJ-1 compared to H-STS cells (*p*<0.01, Figure **2B**).

CgA protein levels (measured by ELISA) also differed in the SI-NEN cell lines (Kruskal-Wallis *p*=0.027) and were particularly higher in the primary (P-STS) than in its metastasis (H-STS, *p*<0.05) (Figure **2D**). The latter observation was mostly consistent with the mRNA results (Figure **2B**). 

The four cell lines all expressed detectable parent CgA bands but expression appeared higher in primary cell lines (Figure **2F**). Since western blot and ELISA use antibodies that bind to CgA at different amino acids, P-STS was not as highly over-expressed as measured by ELISA but was still higher than L-STS. Similar sized bands, consistent with CgA processing (and identified in clinical samples, Figure **2E**) were also noted in the cell lines (Figure **2F**). Interestingly, processing fragments I/II and vasostatin I/II showed increased expression in lymph node and liver metastatic cell line compared to the matched primary (P-STS). Using immunofluorescence, membrane binding of a CgA fragment could not be detected on KRJ-I and H-STS cells (Figure **2G**). We did, however, identify a rapid internalization of this peptide with clear cytoplasmic expression. This is consistent with endocytosis of CgA and suggests a mechanism by which this protein can enter cells and potentially affect signaling pathways.

### CgA processing enzyme expression in small intestinal NENs and cell lines and during proliferation

**Figure 3:**
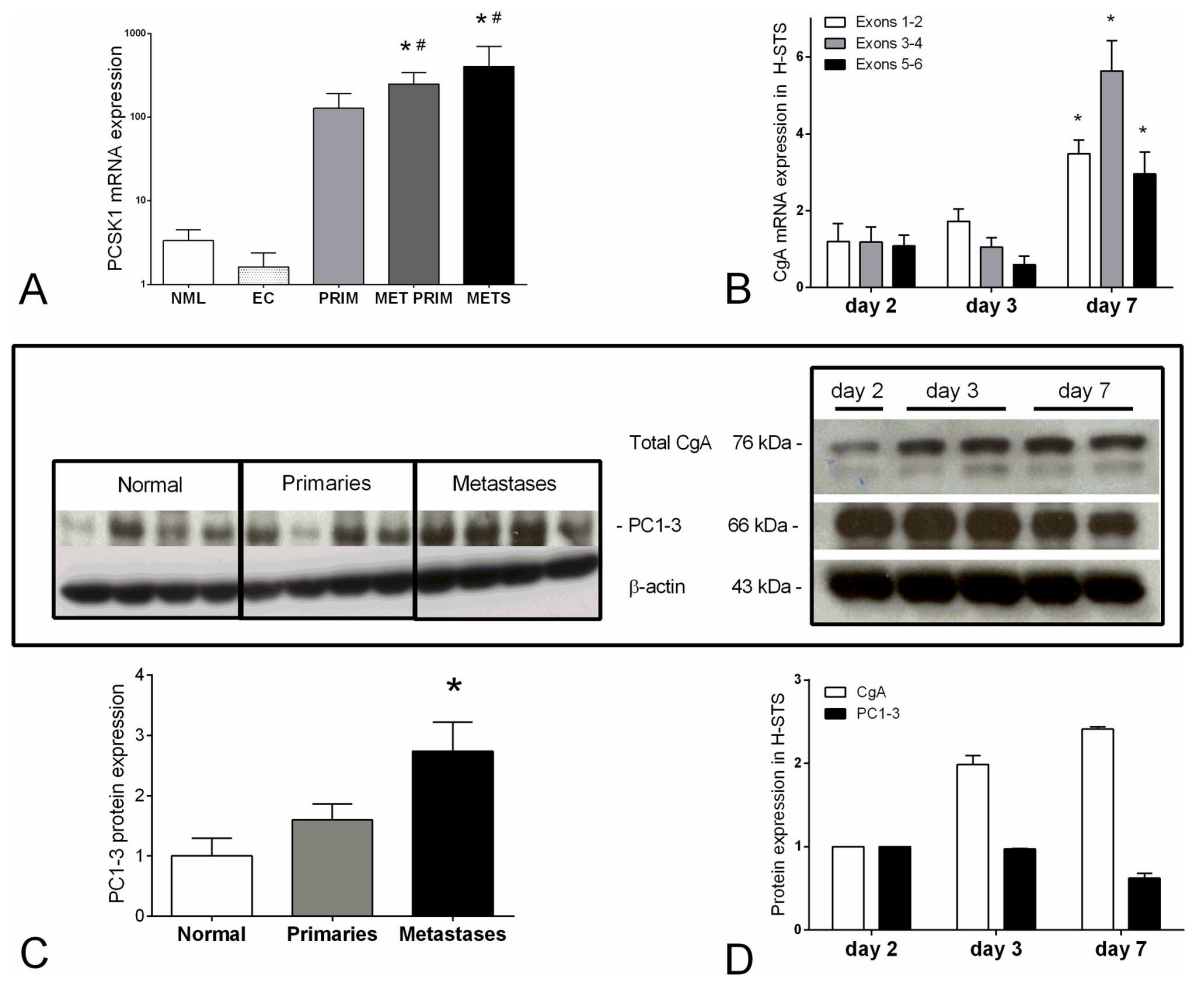
CgA processing enzyme prohormone convertase expression and the effect of tumor growth on CgA and processing. *PCSK1* mRNA expression was increased in SI-NEN metastases (METS) and primaries with metastasis (MET PRIM) compared to normal mucosa (NML, *p<0.05) and normal EC cells (EC, ^#^ *p*<0.05) (**3A**, Kruskal-Wallis *p*=0.0003). Western blot analysis confirmed that protein levels of prohormone convertase 1-3 were elevated in metastases compared to normal mucosa (**3C**,*p<0.05). CgA mRNA (**3B**, 6 exons) and protein (3D) were elevated at the plateau growth phase (day 7) compared to logarithmic growth (day 2) in H-STS cells (*p<0.05). PC1-3 proteins were decreased at day 7 (3D), which can be discussed as one reason for the elevation of total intracellular CgA at this time point. Mean±SEM. PCSK1: prohormone convertase 1.

Given the evidence for different CgA peptide fragments, mRNA and protein expression of the CgA processing enzyme prohormone convertase 1 (*PCSK1*, PC1-3, respectively) was investigated in normal human mucosa, EC cell preparations, localized SI-NENs, primaries with metastasis and liver metastases. Levels were over-expressed in SI-NENs (Figure **3A**, Kruskal-Wallis *p*<0.001) and were increased in metastases compared to localized tumors (Figure **3A**). At a protein level, PC1-3 were higher in metastases compared to normal mucosa (Figure **3C**, *p*<0.05) suggesting this enzyme may be a relevant regulator of CgA processing in SI-NEN metastases.

We next examined the effect of proliferation on CgA and its processing enzyme prohormone converstase. To undertake this, we compared expression in H-STS cells, harvested two and three days after subculture, when cells are in logarithmic growth and after seven days, when cells are in plateau (growth-restricted) phase [20,21,24]. An analysis of CgA mRNA levels identified an up-regulation of all CgA exons at day 7 (Figure **3B**), indicating CgA synthesis may be elevated during slow cell growth in this cell line. In parallel, expression of the predominant 75-80 kDa CgA protein was elevated at day 7 in H-STS compared to day 2 (Figure **3D**), consistent with the mRNA levels. It was further evident that PC1-3 may regulate CgA processing in those cells since prohormone convertase levels were decreased at day 7 when total CgA was increased. We interpret this to demonstrate that alterations occur in CgA processing enzymes as well as in CgA protein itself as the cells proliferate, divide and become quiescent.

### Effects of CgA and its peptide fragments on cell line proliferation and AKT phosphorylation

**Figure 4:**
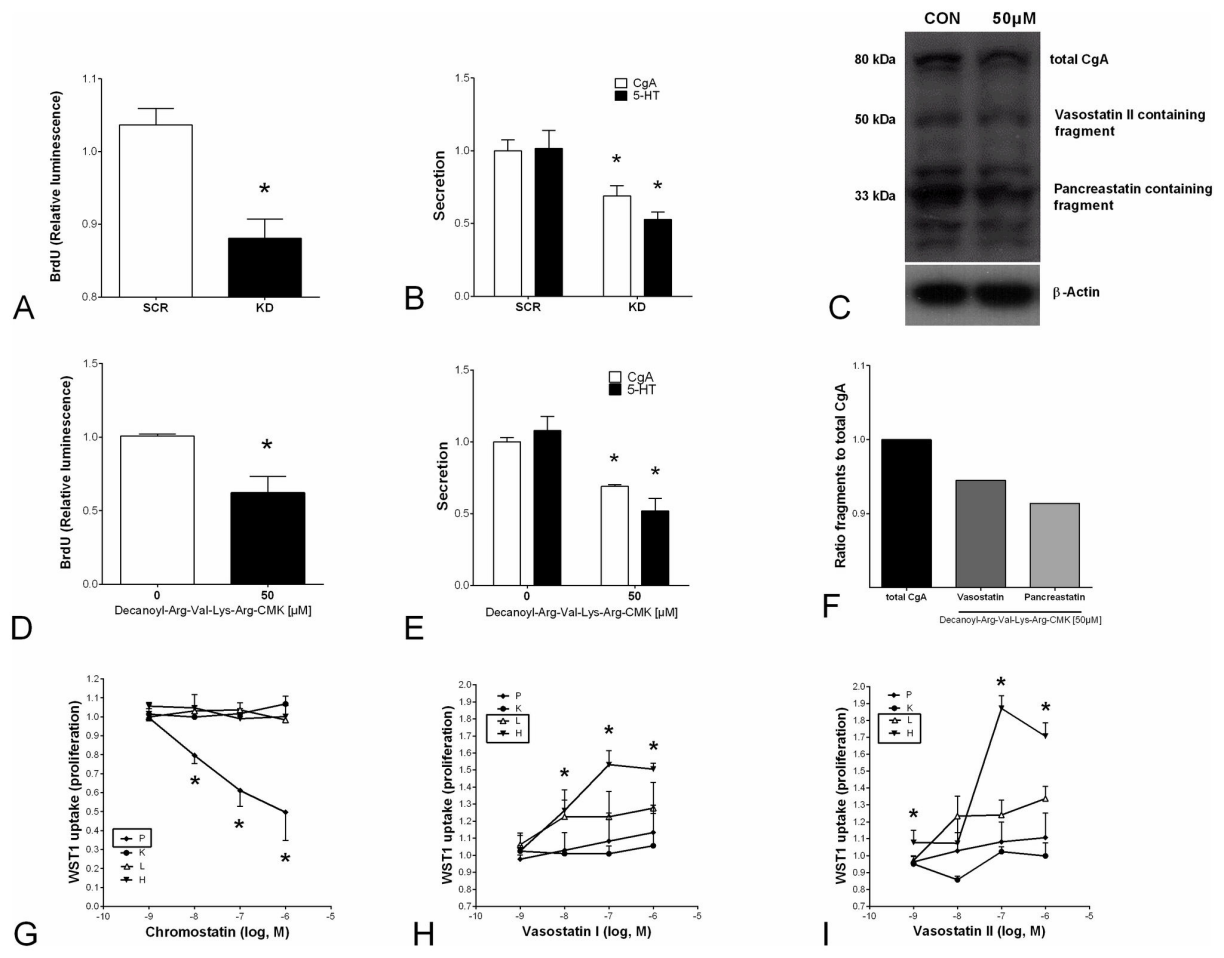
CgA silencing, processing enzyme inhibition and functional analysis of CgA peptides in SI-NEN cell lines. After successfully silencing CgA in H-STS cells (*data not shown*), proliferation was significantly decreased (**4A**, *p<0.05). Secretion of CgA (*p*<0.01) and 5-HT (**4B**, *p<0.05) was significantly reduced following CgA antisense. Inhibition of the CgA processing enzyme prohormone convertase using Decanoyl-Arg-Val-Lys-Arg-CMK also decreased proliferation of H-STS cells (25 µM [*data not shown*] and 50 µM, **4D**, *p<0.05). Additionally, secretion of CgA and 5-HT (**4E**, *p<0.05) was also significantly reduced. Decreases in CgA and its fragments (Vasostatin II and Pancreastatin) after treatment with the prohormone convertase inhibitor were confirmed with western blot (**4C** and **F**). Chromostatin (<20 kDa) was too small to appear on this WB. The fragments Vasostatin I and II significantly stimulated proliferation (up to 60%, *p<0.02) in both metastatic cell lines (L-STS and H-STS, **4H** and **I**, square) but had no effects on the primary tumor cell lines. Chromostatin inhibited the well-differentiated localized NEN cell line proliferation (P-STS, **4G**, square, ~50%, *p<0.05) but not proliferation of the less well-differentiated cell line, KRJ-I. Mean±SEM; *n*=6, CON: control, KD: knockdown, SCR: scrambled, P: P-STS, K:KRJ-1, L: L-STS, H: H-STS. 5-HT: Serotonin.

We next evaluated whether CgA and/or its peptide fragments played a role in regulating proliferation of H-STS cells since those cells exhibited alterations in CgA expression consistent with a regulatory role for this protein. To this end, CgA was successfully silenced (2-fold, *data not shown*), which resulted in a significant decrease in proliferation (87%, *p*<0.05, Figure **4A**). To confirm the efficacy of CgA knockdown on inhibition of cell function, we evaluated CgA and 5-HT secretion. Both were significantly decreased in CgA knockdown cells (31-52%, *p*<0.01, Figure **4B**).

Inhibiting the processing enzyme prohormone convertase using decanoyl-Arg-Val-Lys-Arg- chloromethylketone also resulted in a decrease in proliferation (down to 62% *p*<0.05, Figure **4D**) and was also associated with decreases both in CgA and 5-HT secretion (Figure **4E**). CgA secretion was significantly decreased (down to 69%, *p*<0.01) and 5-HT (down to 49%, *p*<0.05). These physiological alterations, a decrease in CgA and 5-HT secretion, were associated with alterations in CgA processing following inhibition of prohormone convertase (Figure 4C and F).

**Figure 5:**
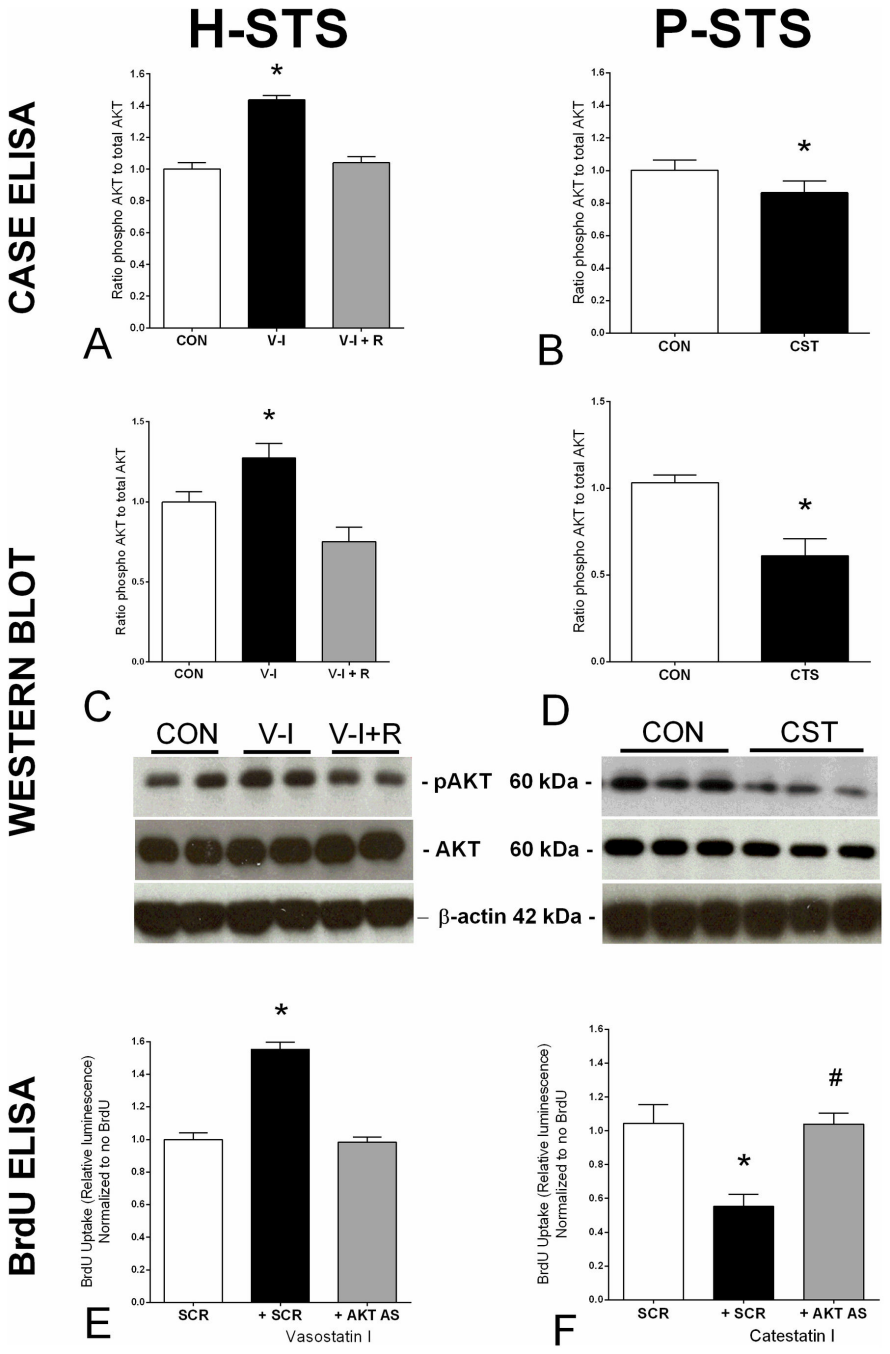
Effect of Vasostatin I and Chromostatin on AKT phosphorylation in metastatic and localized NEN cell lines. Vasostatin I stimulated AKT phosphorylation in the liver metastasis (H-STS) (CASE ELISA: 50%, *p<0.04, western blot: 25%) and could be completely reversed by pre-incubation with RAD001 (**5A/C**, ^#^ *p*<0.01). AKT antisense reversed vasostatin-mediated proliferation (BrdU uptake) (**5E**). In contrast, chromostatin, inhibited AKT signaling in the primary cell line (P-STS) (**5B/D**, ~25%, *p<0.05). AKT antisense reversed chromostatin-mediated inhibition of proliferation (BrdU uptake) (5F). Mean±SD; AS = antisense, CON: control, SCR: scrambled, V-I: vasostatin I, R: RAD001, CST: chromostatin.

Thereafter, we performed functional analysis of eight candidate CgA peptides in the four SI-NEN cell lines. In initial studies, we have identified Cy5-labeled CgA immunostaining of KRJ-I and H-STS cells, which was internalized (Figure **2G**). This suggests that a CgA/peptide mediated effect may represent a process of intracellular activation as opposed to the more classical, membrane-bound receptor mechanism. In the current study, C-terminal derived fragments had little effect on cell line proliferation. Pancreastatin, fragment 1, fragment 2, catestatin and WE14 had no proliferative effect (*data not shown*), while chromostatin had an anti-proliferative effect on the primary cell line P-STS (Figure **4G**). In contrast, N-terminal and the middle fragments did affect cell proliferation. Specifically, vasostatin I and II significantly stimulated proliferation (up to 60%, *p*<0.02) in both metastatic cell lines (L-STS and H-STS) but had no effects on primary tumor cell lines (P-STS and KRJ-1) (Figure 4H and I). This effect was associated with AKT phosphorylation (CASE ELISA: 50%, *p*<0.04; western blot: 25%) (Figure **5A, C**) and was completely reversed by pre-incubation with the mTOR inhibitor RAD001 (*p*<0.01). AKT antisense also was able to reverse vasostatin-mediated BrdU uptake (proliferation) (Figure **5E**). In contrast, chromostatin, inhibited localized cell proliferation (CASE ELISA: ~20%, *p*=0.03) (Figure **5B**) as well as AKT phosphorylation (~50%; Figure **5D**) in the localized P-STS cell line, effects that were reversed by RAD001. AKT antisense reversed chromostatin-mediated BrdU inhibition (Figure **5F**). These growth regulatory effects signaled predominantly via Ser473 phosphorylation, a known regulator of SI-NEN cell line proliferation [33].

## Discussion

Chromogranin A is a pro-hormone that is differentially processed into peptides that regulate a range of biological functions including cell proliferation, angiogenesis and hormonal secretion [36]. The current study identified that CgA mRNA and proteins were differently expressed in SI-NEN progression, from normal EC cells to metastatic cancer, and that advanced disease was associated with gain of specific CgA fragments (largely middle and N-terminal peptides) that were effectors of growth via AKT/mTOR signal cross-activation. 

CgA mRNA and proteins/peptides were, in comparison to normal mucosa and EC cells, increased in SI-NENs, which were also characterized by the expression of CgA processing fragments consistent with N-terminal fragments. The mechanisms underlying the altered expressions may reflect either transcriptional or post-translational mechanisms. CgA transcription is well-known to be regulated via CRE sites in the promoter [37]. We have undertaken a microarray screen of SI-NENs and identified CRE-mediated signaling as a common pathway in these tumors [38]. It is possible that alterations in growth signaling pathways that signal through cAMP are associated with the alterations in CgA transcription identified in SI-NEN metastases in this study. 

While CgA is commonly considered a marker of tumor load and plasma levels are related to progression free survival in GEP-NENs [19,39], this most likely reflects tumor mass *per se* [40,41]. In the current study, we identified that metastases expressed less CgA than primary tumors (when normalized to total protein) and that the two metastatic cell lines we investigated exhibited lower levels of CgA mRNA and protein compared to cell lines derived from primary tumors. We postulate that alterations in CgA expression, particularly at the level of post-translational processing may be a feature of more malignant NENs and may play a role in regulating proliferation. CgA has been identified to play a role in preventing tumor cell seeding and progression in a mouse model of breast adenocarcinoma [42], suggesting that elevated CgA levels (perhaps of specific fragments – this was not assessed in the study) may have an inhibitory role in neoplastic development. Our observations suggest that differences occur in the processing and the production of specific fragments that may provide an important, under-examined mechanism for these processes. 

One of the CgA fragments that was differentially processed during SI-NEN metastasis was vasostatin I/II which is recognized to have vasoconstrictive effects on small and medium resistance vessels in cardiovascular system [43]. Although considered a candidate factor in cancer gene therapy [44,45], cell adhesion, spreading and cellular invasion, vasostatin enhanced malignant behavior in mice implanted with vasostatin-expressing BON cells through mechanisms that involved cell cycle regulation (i.e. p27^*Kip1*^) [46]. In the current study, vasostatin I, selectively stimulated proliferation of the metastatic cell lines, L-STS and H-STS, through AKT/mTOR activation, a known regulator of p27^*Kip1*^ [47]. These vasostatin-mediated effects were modulated by phosphorylation at Ser473, recognized as the phosphorylation site associated with growth-regulatory signaling in SI-NEN cell lines and neoplasms [33]. These effects occurred at clinically relevant concentrations; plasma CgA levels in patients affected with SI-NEN liver metastases range from 10^-4^ to 10^-7^M [19]. The two localized cell lines, KRJ-I and P-STS, were not affected by these peptides. Vasostatin-mediated proliferation appeared to reflect a gain of function consequence of metastasis, an effect that we consider due to differential CgA processing. These proliferative effects are most likely due to intracellular activation of the AKT/mTOR pathway, as we did not identify a membrane-bound receptor for CgA. Since CgA peptide effects, particularly, vasostatin, has been demonstrated to occur through internalization and activation of intracellular proteins in HUVEC cells [48], we postulate that internalization of peptides may affect signaling pathways in SI-NENs in a non-membrane receptor manner. 

In contrast to vasostatin, chromostatin inhibited proliferative activity in P-STS cells through inhibition of AKT phosphorylation, which is, to the best of our knowledge, the first identification that this CgA fragment has an anti-proliferative effect in NENs.

An emerging area of interest is regulation of pro-hormone processing enzymes, either spatially or at the level of cellular expression, that may play an important role in cleavage and secretion of hormones [49]. The classical prohormone convertases (PC1-3) selectively process precursors e.g. CgA to pancreastatin, whose products are stored in secretory granules [14]. Variation in *PC1* and *PC2* mRNA expression has been suggested to play distinct roles in the activation of brain pro-proteins, particularly CgA, while expression of this enzyme itself appears regulated at a CRE-level, at least in the pancreatic NEN cell line BON [50]. In the current study, *PC*_*1/2*_ mRNA was identifiable in normal EC cells, but was over-expressed in SI-NENs while *PC1* itself was significantly elevated in metastases. Inhibiting this enzyme resulted in a decrease in H-STS proliferation as well as down-regulation of CgA and 5-HT secretion demonstrating the sensitivity of post-translational modifications within the neoplastic CgA system. 

In conclusion, we identified that CgA was differentially regulated in primary and metastatic SI-NEN cell lines, which exhibited elevated (compared to normal EC cells) but different patterns of CgA fragments with different functions. Specifically, N-terminal fragments stimulated metastatic NEN cell line proliferation while middle fragments inhibit localized tumor cell proliferation via the AKT/mTOR pathway. The presence and over-expression of prohormone convertases 1-3 in neuroendocrine neoplasms suggested that CgA was altered at a post-translational stage via this enzymes. It therefore seems likely that the CgA fragment pattern may be of value in evaluating the biologic behavior of NENs and the region-specific antibodies may be of potential use when seeking to identify individual malignant tumor phenotypes.
